# Combinations of Small RNA, RNA, and Degradome Sequencing Uncovers the Expression Pattern of microRNA–mRNA Pairs Adapting to Drought Stress in Leaf and Root of *Dactylis glomerata* L.

**DOI:** 10.3390/ijms19103114

**Published:** 2018-10-11

**Authors:** Yang Ji, Peilin Chen, Jing Chen, Kayla K. Pennerman, Xiaoyu Liang, Haidong Yan, Sifan Zhou, Guangyan Feng, Chengran Wang, Guohua Yin, Xinquan Zhang, Yuanbin Hu, Linkai Huang

**Affiliations:** 1 Sichuan Animal Science Academy, Chengdu 610066, China; jiyang221@163.com (Y.J.); liangxiaoyucao@163.com (X.L.); huyuanbin2233@163.com (Y.H.); 2 Department of Grassland Science, Faculty of Animal Science and Technology, Sichuan Agricultural University, Chengdu 611130, China; chenpeilin1994@outlook.com (P.C.); chenjing123ing@163.com (J.C.); swanchou93@163.com (S.Z.); b20152501@stu.sicau.edu.cn (G.F.); wlr6299@sina.com (C.W.); Zhangxq@sicau.edu.cn (X.Z.); 3 Department of Plant Biology, Rutgers, The State University of New Jersey, New Brunswick, NJ 08901, USA; kkp63@scarletmail.rutgers.edu (K.K.P.); guohuayin1997@gmail.com (G.Y.); 4 Department of Horticulture, Virginia Tech, Blacksburg, VA 24061, USA; yanhd@vt.edu

**Keywords:** orchardgrass, drought stress, miRNA, degradome, transcriptome

## Abstract

Drought stress is a global problem, and the lack of water is a key factor that leads to agricultural shortages. MicroRNAs play a crucial role in the plant drought stress response; however, the microRNAs and their targets involved in drought response have not been well elucidated. In the present study, we used Illumina platform (https://www.illumina.com/) and combined data from miRNA, RNA, and degradome sequencing to explore the drought- and organ-specific miRNAs in orchardgrass (*Dactylis glomerata* L.) leaf and root. We aimed to find potential miRNA–mRNA regulation patterns responding to drought conditions. In total, 519 (486 conserved and 33 novel) miRNAs were identified, of which, 41 miRNAs had significant differential expression among the comparisons (*p* < 0.05). We also identified 55,366 unigenes by RNA-Seq, where 12,535 unigenes were differently expressed. Finally, our degradome analysis revealed that 5950 transcripts were targeted by 487 miRNAs. A correlation analysis identified that miRNA *ata-miR164c-3p* and its target heat shock protein family A (HSP70) member 5 gene *comp59407_c0* (*BIPE3*) may be essential in organ-specific plant drought stress response and/or adaptation in orchardgrass. Additionally, Gene ontology (GO) and Kyoto encyclopedia of genes and genomes (KEGG) analyses found that “antigen processing and presentation” was the most enriched downregulated pathway in adaptation to drought conditions. Taken together, we explored the genes and miRNAs that may be involved in drought adaptation of orchardgrass and identified how they may be regulated. These results serve as a valuable genetic resource for future studies focusing on how plants adapted to drought conditions.

## 1. Introduction

Drought stress is a recurring phenomenon that negatively impacts human-made and natural environments [1,2,3]. Droughts have become more frequent and severe in the twenty-first century, making the recovery time for an ecosystem shorter. Long-lasting incomplete recovery states may become more firmly established, with adverse consequences for the land carbon sink [4,5]. The combination of droughts and global warming is likely to have a greater effect on the environment and may induce other abiotic stresses [6]. The physiological acclimation of individual plants plays a role in buffering the impact of drought [4], thus, it is crucial to improve drought resistance of plants for further adaptation.

Studies have elucidated molecular mechanisms underlying drought resistance in plants. The expression of genes involved in drought response change to maintain homeostasis of the cells [7]. For example, osmotic stress, a signal of drought stress, can activate genes in the mitogen-activated protein kinase (MAPK) and calcium-dependent protein kinase (CDPK) pathways, and promote or inhibit expression of downstream genes such as *WRKY* [8] and *bHLH* [9] transcription factors. MicroRNA (miRNA), an endogenous noncoding RNA that is about 22 nt [10], is a crucial post-transcriptional regulator that targets mRNAs to control mRNA degradation or repress the translation [11]. In plants, miRNAs contribute to abiotic stress tolerance [12], and the potential regulation patterns of miRNAs for drought stress have been reported in wheat (*Triticum aestivum* L.) [13], rice (*Oryza sativa*) [14], tobacco (*Nicotiana tabacum* L.) [15], arabidopsis (*Arabidopsis thaliana* (L.) Heynh.) [16], tomato (*Lycopersicon esculentum* Mill.) [17], sugarcane (*Saccharum officinarum*) [18], sorghum (*Sorghum bicolor* (L.) Moench) [19], barley (*Hordeum vulgare* L.) [20], and cotton (*Gossypium* spp.) [21]. The conserved miRNAs *miRNA156*, *miRNA166*, *miRNA169*, and *miRNA396* are expressed in multiple plants responding to drought. Some miRNAs have specific functions under drought stress. For example, *miR156* increases the drought tolerance in alfalfa (*Medicago sativa*) by silencing a squamosa promoter binding protein-like (SPL) gene [22], and a rice *miR166* knockdown mutant shows differences in leaf rolling and adjusts stem xylem development, improving drought tolerance [23]. *miR169* functions in reducing stomatal opening, lowering leaf water loss, and decreasing transpiration rate to enhance drought tolerance in tomato [17]. To better understand the functions of miRNAs in drought response and/or adaptation, it is essential to identify the targets of each miRNA and understand the expression patterns of these targets. Traditional computational target prediction tools generally select evolutionarily conserved miRNA binding sites. False positives are fairly high when using these tools, making the prediction results usually biologically irrelevant across different species [24]. Degradome sequencing, or parallel analysis of RNA ends (PARE), involves high-throughput sequencing with Illumina’s SBS technology to modify 5′-rapid amplification of cDNA ends (RACE) [25,26]. miRNAs cause endonucleolytic cleavage of mRNAs, making the degrading products complementary to the mRNAs [27], and degradome-seq allows for the identification of miRNA targets without the use of prediction software. Recently, degradome sequencing has been used to find precise miRNA-target relationships in various plants species, including liverwort [28], soybean [29], and tomato [30].

Orchardgrass (*Dactylis glomerata* L.) belongs to the Poaceae family and is the fourth most crucial forage grass around the world [31]. It is a perennial cool-season grass that is widely spread in Middle Eastern regions [31,32]. As an economically important forage, orchardgrass has been widely cultivated for hay or silage production and grazing, because of its high adaptability, nutrition, shade tolerance, and biomass production [33,34]. In a previous study, some orchardgrass accessions were able to tolerate severe drought and still produce high yields [35]; however, the drought resistance mechanism remains unclear. Here, we use the drought-tolerant genotype of orchardgrass named “Baoxing”, which originates from Baoxing county in Sichuan [36], and we integrated transcriptome, miRNAome, and degradome results after water deficit treatment to identify drought-adaptive genes and miRNAs in leaf and root. In addition, we aimed to find potential regulation patterns of miRNA–target pairs. This study will help drought resistance breeding of crops, and serves as fundamental gene-level research that can speed up recovery time of ecosystems between severe and frequent droughts.

## 2. Results

### 2.1. Transcriptome Sequencing of Orchardgrass under Drought Treatment

To understand the gene expression profiles, we performed RNA sequencing on RNA extracted from leaf and root under control and 18 d drought conditions (CK_L, D18d_L, CK_R, D18d_R) using Illumina Hiseq2500. Transcriptome sequencing generated 6.5–10.31 Gb sequencing data for the eight samples, and the total RNA yield for each sample ranged from 36,675 to 42,652 (Table S2). After quality control, 55,366 unigenes were assembled from the remaining high-quality reads, with an average length of 610 bp. Of the 55,366 unigenes, 34,077 unigenes (61.55%) were predicted to have coding sequences.

**Figure 1:**
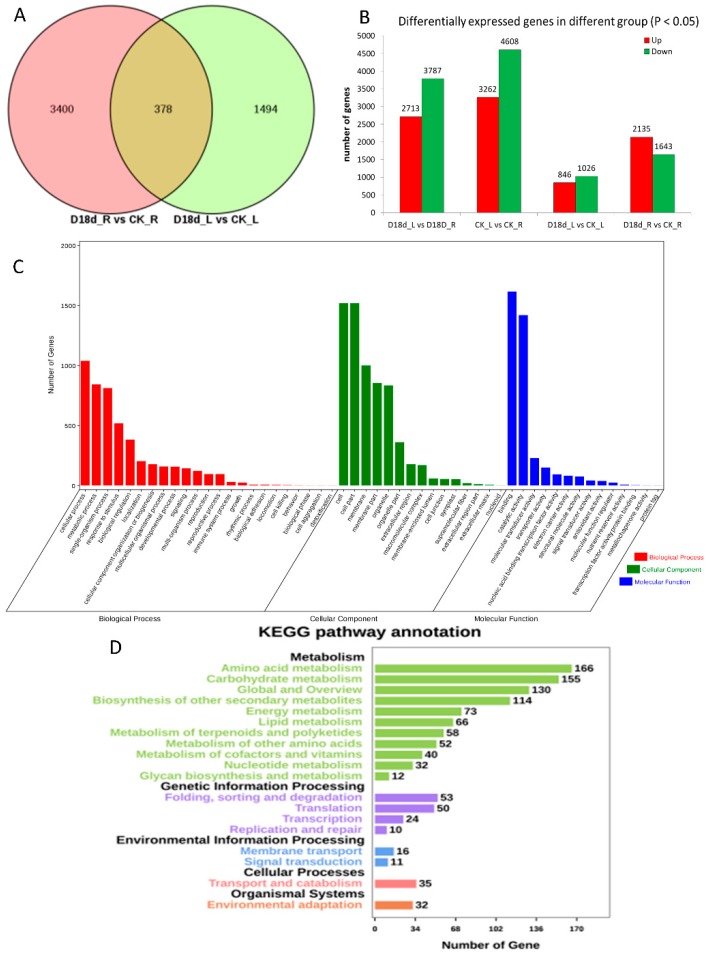
Differentially expressed genes (DEGs) in orchardgrass treated with drought stress. (**A**) Venn diagram of DEGs from root and leaf after drought treatment; pink color represent to comparison of drought treat root to controlled root, light-yellow color represent to comparison of drought treat leaf to controlled leaf; (**B**) column graph of DEGs in different treatment groups showing both upregulated and downregulated genes; (**C**) gene ontology annotation for all 5272 DEGs; (**D**) Kyoto encyclopedia of genes and genomes pathway annotation for all 5272 DEGs.

A total of 5272 differentially expressed genes (DEGs) were identified in leaf and/or root treated with 18-day drought stress (default threshold: |log2foldchange| ≥ 1, *p* < 0.05; Figure 1A). We identified 1872 DEGs in leaves and 3778 DEGs in roots after drought treatment; 378 DEGs were common to both leaf and root. Among the four comparisons, only the D18d_R vs. CK_R comparison had more upregulated DEGs (2135) than the downregulated DEGs (1643) (Figure 1B). The gene ontology (GO) annotation of the 5272 DEGs that adapted to drought stress had biological functions such as binding, catalytic activity, cell, cell part, cellular process, metabolic process, and response to stimulus (Figure 1C). The DEGs were also involved in important pathways, including amino acid metabolism, carbohydrate metabolism, and environmental adaption (Figure 1D). These pathways may be induced by drought stress. Separate annotations of DEGs in leaf and root did not result in large differences in the proportion of the different terms (Figure S1).

**Figure 2:**
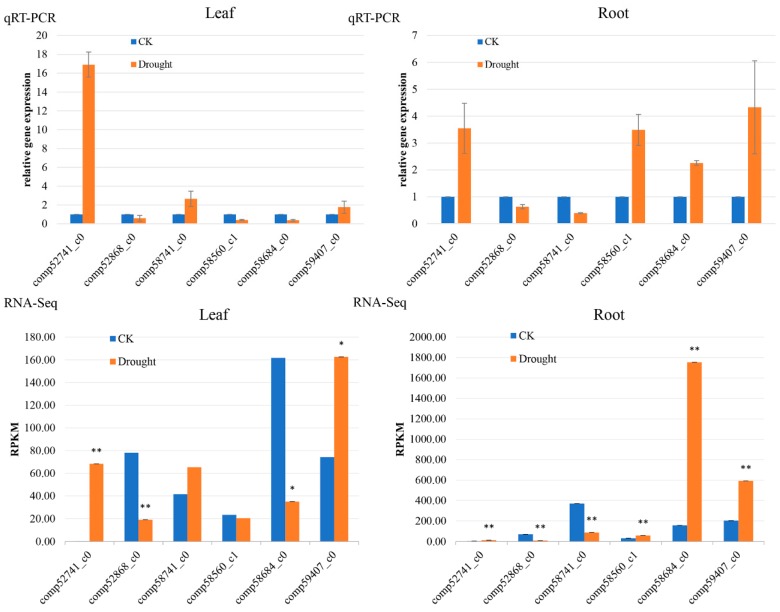
qRT-PCR verification of RNA-Seq analysis of gene expression. Blue columns stand for control treatment, and orange columns stand for drought treatment. qRT-PCR: Quantitative real time polymerase chain reaction; CK: controlled samples; Drought: drought treated samples; RPKM: Reads Per Kilobase per Million mapped read. * *p* < 0.05; ** *p* < 0.01.

To validate the RNA-Seq results, six genes were chosen for quantitative real-time polymerase chain reaction (qRT-PCR) analysis. The six genes were *comp52741_c0* (protein argonaute 2 gene: *AGO2*), *comp52868_c0* (MYB-related protein gene: *MYBAS2*), *comp58741_c0* (NAC domain-containing protein 74 gene: *NAC74*), *comp58560_c1* (protein kinase R (PKR)-like endoplasmic reticulum kinase 2 gene: *PERK2*), *comp58684_c0* (sugar transport protein 13 gene: *STP13*), and *comp59407_c0* (luminal-binding protein 3 gene: *BIPE3*) (Figure 2). *AGO2* and *BIPE3* were upregulated in both organs after drought stress, while *MYBAS2* was significantly downregulated. *NAC74*, *PERK2*, and *STP13* showed opposite expression trends in leaf and root after drought treatment. In leaf, *NAC74* showed increased expression under drought stress, while *PERK2* and *STP13* showed decreased expression. In root, *NAC74* expression decreased, while *PERK2* and *STP13* expression increased under drought stress. The qRT-PCR results were in accordance with the RNA-Seq results.

### 2.2. Construction of Global Small RNAs Library

To elucidate the abundance of miRNAs after adaptation to drought stress, we sequenced the small RNAs from the eight samples used to generate the RNA-seq library. In total, 10,170,218–18,715,594 raw and 2,710,475–8,805,921 valid reads were obtained (Table S3). The read lengths ranged between 20–24 nt, consistent with the cleavage features of Dicer enzymes (Figure S2) [10]. We detected 486 conserved miRNAs, including 14 known miRNAs, and 33 miRNAs novel to miRBase (Table S4). Following the regular pattern of miRNA lengths in plant, 21 nt sequences comprised the largest proportion (35.36%) of unique miRNAs (Table S5). We predicted 487 miRNAs with 5950 targets, totaling 12,663 miRNA-target pairs (Table S6).

### 2.3. Degradome Sequencing Analysis

To narrow down the prediction range of miRNA-target pairs and to avoid false positives caused by the prediction software, we conducted degradome sequencing analysis. A total of 34,924,858 mappable reads were generated after filtering 35,065,665 degradome raw reads. The eight RNA sequence datasets, including 55,366 transcripts, were utilized to detect miRNA cleavage sites of orchardgrass. Of the transcripts, 45,765 (82.66% of the input transcripts) mapped to 21,363,848 degradome reads, resulting in a 60.93% mapping ratio (Table S7). Based on the degradome sequence data, 347 miRNAs (including 328 conserved and 19 novel miRNAs) with 799 target transcripts were verified to form 1449 miRNA–target pairs. In our result, the numbers of potentially cleaved targets in degradome categories 0–4 were 116, 7, 798, 168, and 360, respectively (Table S8).

**Figure 3:**
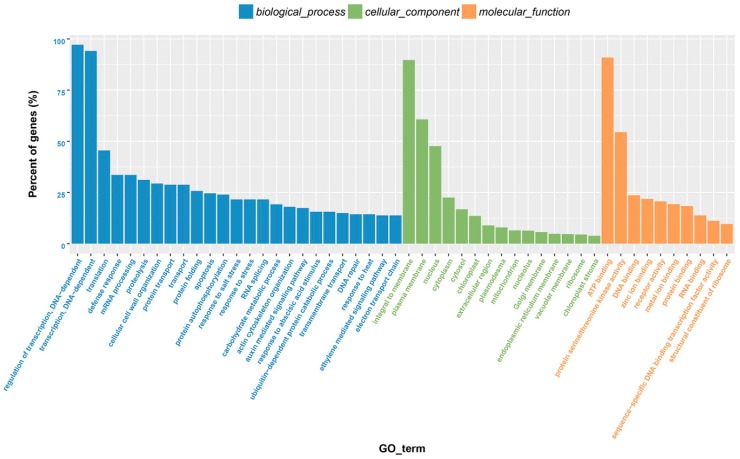
GO annotation of the genes targeted by miRNAs.

In sum, 487 genes that were targeted by the identified miRNAs were annotated through GO analysis. The targets were classified into 346 biological process, 285 molecular functions, and 134 cellular components. Of the 25 categories of biological processes, “regulation of transcription and DNA-dependent”, and “transcription, DNA-dependent” were the most abundant. “Regulation of transcription and DNA-dependent” was also the most enriched group among all GO categories. For cellular component, the most frequent categories were “nucleus”, “integral to membrane”, and “plasma membrane”. Among the molecular function categories, “ATP binding”, which was assigned to 129 unigenes, constituted the most abundant molecular function category (Table S9), followed by “protein serine/threonine kinase activity” (Figure 3).

**Figure 4:**
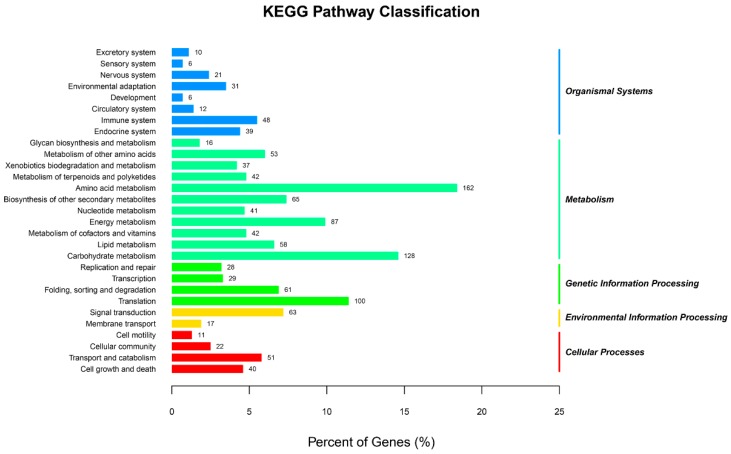
KEGG pathway classification of genes targeted by miRNAs.

We next conducted the KEGG analysis, and annotated 175 predicted targets to 223 different pathways. The target genes mostly fell under the KEGG class metabolism. “Amino acid metabolism”, within the metabolism class, was associated with 162 unigenes, making it the most abundant group, indicating the crucial role of amino acids under drought stress. The pathways “immune system” in Organismal Systems, “translation” in Genetic Information Processing, “signal transduction” in Environmental Information Processing, and “transport and catabolism” in Cellular Processes, were the most represented groups among the different classes (Figure 4).

### 2.4. Correlation Analysis of miRNA–Target Pairs

**Figure 5:**
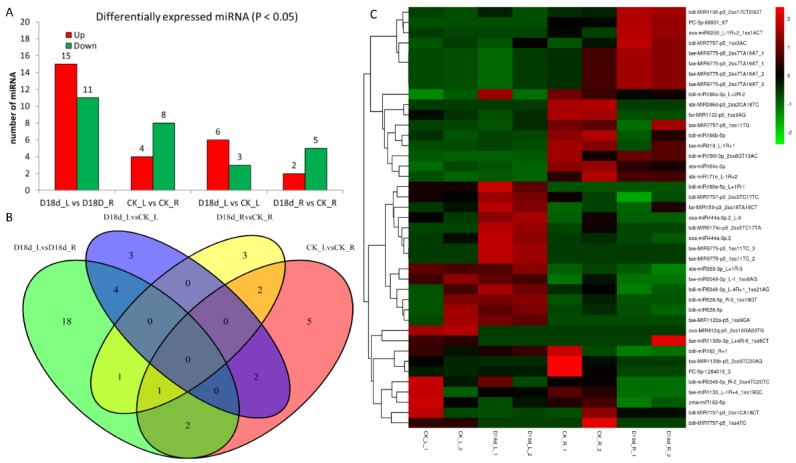
Analysis of differentially expressed miRNAs (DEmiRs) in orchardgrass exposed to drought. (**A**) Barplot of DEmiRs from different groups with significance threshold of 0.05; (**B**) Venn diagram of different comparison groups (*p*-value < 0.05); green color represent to comparison of drought treated leaf to root; purple color represent to comparison of drought treat leaf to controlled leaf; yellow color represent to comparison of drought treat root to controlled root; pink color represent to comparison of controlled leaf to root; (**C**) expression heatmap of the 41 DEmiRs (the samples names are shown at the bottom). The original expression values were normalized by Z-score normalization. With color changes from green to red, the absolute signal intensity ranges from −2.0 to +2.0.

Differentially expressed miRNAs (DEmiRs) were analyzed to identify key DEmiRs that may be involved in drought resistance. In total, 41 miRNAs (*p*-value < 0.05) showed the following differential expression patterns (Table S10; Figure 5A): between CK_L and CK_R, there were four upregulated and eight downregulated miRNAs; between D18d_L and D18d_R, there were 15 upregulated and 11 downregulated miRNAs; between D18d_L and CK_L, there were six upregulated and three downregulated miRNAs; and between D18d_R and CK_R, there were two upregulated and five downregulated miRNAs. There were nine and seven DEmiRs when comparing D18d_L vs. CK_L and D18d_R vs. CK_R, respectively, though no intersection was found between these two comparisons. However, three miRNAs (ata-miR393-3p_L+1R-3; ata-miR164c-3p; PC-3p-68901_67) were significantly differently expressed in both D18d_L vs. D18d_R and CK_L vs. CK_R comparisons (Figure 5B,C). ata-miR393-3p_L+1R-3 was upregulated among the two comparisons, whereas ata-miR164c-3p and PC-3p-68901_67 (a novel miRNA) were downregulated. In addition, ata-miR164c-3p was found to be involved in the comparison D18d_R vs. CK_R. Drought stress causes significantly more damage to the leaves than roots of plants, partly due to the accumulation of panniculus peroxidation product malonic dialdehyde (MDA) in leaves and the fast-growing membrane permeability of leaves [37]. Thus, we inferred that ata-miR164c-3p may play a key role in drought tolerance of root or may be involved in adaptation to drought tolerance.

**Figure 6:**
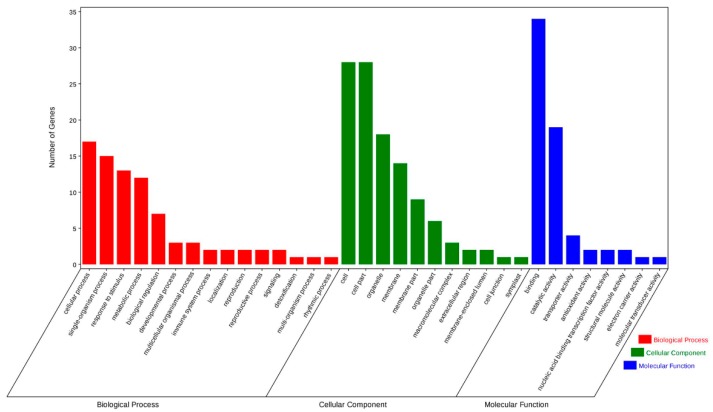
GO categories of the target genes for 25 DEmiRs.

To identify the expression profile at the transcriptional level, we conducted a combination analysis of degradome data and data of both mRNA and miRNA. Of the 41 DEmiRs, 25 miRNAs were predicted to target 74 different mRNAs forming 107 miRNA–target pairs, and those targets were annotated to 34 different GO terms (Figure 6).

**Figure 7:**
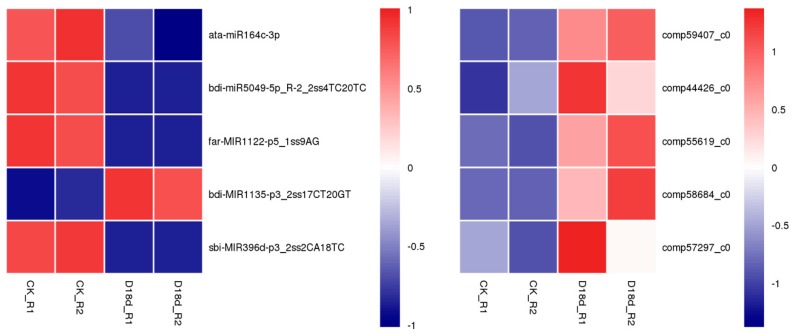
Expression of significantly expressed DEmiRs and their target DEG in the roots. The original expression values were normalized by Z-score normalization. Changes in color from blue to red denote changes in absolute signal intensity from −2.0 to +2.0.

Of the 107 pairs, nine pairs (seven DEmiRs with nine targeted DEGs) were found when comparing CK_L vs. CK_R, among them, only three pairs showed reversed regulation patterns (Table 1). When focusing on the regulation pattern adapting to drought stress, we did not identify any DEmiR-DEG pairs with significant differential expression in leaves, while we identified seven pairs (five DEmiRs with five targeted DEGs) that were significantly differentially expressed in roots. Six of these pairs displayed reversed expression pattern under drought condition (Figure 7). For example, *ata-miR164c-3p* was significantly downregulated under 18-day drought stress, while its target, *comp59407_c0* (*BIPE3*), was significantly upregulated.

The target gene comp42625_c0 that was detected in the comparison CK_L vs. CK_R was involved in three KEGG pathways: “phenylpropanoid biosynthesis” (ko00940), “phenylalanine metabolism” (ko00360), and “methane metabolism” (ko00680). Focusing on drought effects in the root, there were two targets annotated to different pathways: comp59407_c0 (*BIPE3*) was involved in “protein export” (ko03060) and comp55619_c0 was involved in “flavonoid biosynthesis” (ko00941) (Table 2).

## 3. Discussion

### 3.1. Small RNAs and Their Expression Pattern in Plants under Drought Stress

As crucial post-transcriptional gene regulators, miRNAs play important roles in plants responding to drought stress, as evidenced by the wide range of drought-related genes they targeted by miRNAs, such as *ARF*, *DREB*, *WRKY*, *NAC*, *TCP*, *MYB*, and *GRAS* family transcription factors, as well as abscisic acid and dehydrin-related genes [38,39,40,41,42,43,44]. Drought-related miRNAs have been identified in wheat [45], tobacco [15], sorghum [46], sugarcane [18], potato [47], barley [20], tomato [30], and cotton [21]. However, the function of miRNAs remained unclear in *Dactylis glomerata* L. (orchardgrass), the fourth most important forage grass in the world [48]. Using high throughput sequencing, we identified the role of miRNAs during adaptation to drought in both aboveground (leaf) and belowground (root) organs of orchardgrass.

We identified 486 conserved and 33 novel miRNAs, 41 of which showed differential expression patterns among comparisons. This differed from a study in tomato, where authors found that drought stress significantly changed the expression patterns of 11 miRNAs in all organs of both sensitive and tolerant genotypes [30]. In our study, there were no common differentially expressed miRNAs between control and treatment groups of either leaf or root. However, we found three miRNAs (*ata-miR393-3p_L+1R-3; ata-miR164c-3p; PC-3p-68901_67*) with different expression patterns between leaf and root, regardless of drought stress.

Among the three miRNAs, *ata-miR393-3p_L+1R-3* was upregulated in leaves compared roots. The MIR393 family has been widely studied in model plants and is involved in auxin response [49,50]. Gao and colleagues generated overexpressed *osa-miR393* transgenic rice and *Arabidopsis thaliana*, which are more sensitive to salt and alkali stresses compared to wild plant [51]. Overexpression of *miR393* resistance genes enhances salt tolerance in *Arabidopsis* [52]. These studies illustrate that *miR393* has negative impacts on plant salt-alkali stress tolerance. Some studies report that *miR393* influences root growth and adventitious root numbers by altering auxin sensitivity [53]. It remains unclear whether *miR393* is involved in drought response; however, *miR393* plays a role in organ-specific regulation and abiotic stress tolerance.

MicroRNAs *ata-miR164c-3p* and *PC-3p-68901_67* were downregulated in two comparisons; *ata-miR164c-3p* was also significantly downregulated in drought-stressed roots compared to the control. *miR164* is a plant specific miRNA family that was discovered in arabidopsis and has two forms (*ath-miR164a* and *ath-miR164b*). Two homologous sequences are found in the rice genome database (*osa-miR164a* and *osa-miR164b*) [54]. The NAC transcription factor family is the main target of *miR164*. In rice, NAC is a negative regulator for drought response and *miRNA164* improves drought tolerance by targeting NAC genes [55]. In previous studies, *miR164* associates with drought response in foxtail millet [56], indica rice [57], and cassava [58]. Therefore, we inferred that *miR164* may play a crucial role in responding to drought stress or is involved in adaptation to water deficiency in orchardgrass. We found that *ata-miR164c-3p* expression was significantly lower in leaves compared to roots. In a previous study [37], the damage on orchardgrass leaf blades was far more serious than roots under constant drought stress, and *ata-miR164c-3p* may regulate the different tolerance of these two organs. In the comparison between D18d_R and CK_R, *ata-miR164c-3p* was also downregulated. This may have resulted from constant drought stress inhibiting its expression.

### 3.2. Target Parsing of Small RNAs in Orchardgrass

Degradome analysis was performed to further explore the involvement of miRNAs in drought adaptation. Numerous target genes were determined for the known and novel miRNAs. We found 347 miRNAs (including 328 conserved 19 novel miRNAs) with 799 target transcripts. Most targets for differentially expressed miRNAs were detected, and many of the targets were known regulators of drought adaptation, such as *AP2/ERF* [59], *HD-Zip/bZIP* [60], *NAC* [61], *MYB* [62], *WRKY* [40], *HSP* [63], and *DREB* [64] transcription factors. We further verified the expression of some drought stress-related genes and transcription factors (Figure 2). Consistent with the transcriptome results, *AGO2* (*comp52741_c0*) showed increased expression under drought conditions in both organs, which associates with the miRNA assembling processes under abiotic stress, in order to induce mRNA degradation [65]. Similarly, the HSP protein BIPE3 was also significantly upregulated in both organs under drought treatment, consistent with previous studies that showed the involvement of HSPs in drought stress adaptation [66]. Conversely, *NAC74* and *MYBAS2* genes were downregulated in root, but did not show significant changes in leaf under drought stress. This differed from a previous study that showed increased expression of *NAC* genes in soybeans under drought stress [61]. These results suggest that the expression pattern of *NAC* and *MYBAS2* genes may vary among species or specific homologs.

The common annotations of the 487 targets identified from the degradome were “regulation of transcription, DNA-dependent”, “DNA binding”, and “nucleus”. Among the 129 unigenes, “ATP binding” was the most abundant annotation, although it was not significantly differentially expressed. ATP binding protein genes might be induced by drought stress, as previously identified in sugarcane [67].

“Antigen processing and presentation” was the most enriched pathway in the degradome annotation (Figure S3), and it was downregulated under drought stress. In this study, we found increased expression of heat shock protein 70 (*HSP70)* family genes which can associate with peptides and interact with antigen presenting cells, suggesting that the pathway “antigen processing and presentation” was downregulated after HSP70s interacted with the relevant antigens [68]. There were three genes (*comp51164_c1, comp57333_c0* and *comp49277_c0*) involved in this pathway, and two of them (*comp51164_c1, comp57333_c0*) were reversely regulated by *miR169*. Gene *comp51164_c1* was a *NFYA5* gene which was annotated to be involved in response to water deprivation and other resistance-related functions, such as the abscisic acid-mediated signaling and blue light signaling pathways. Gene *comp57333_c0*, a *NFYA1* gene, was annotated to be involved in regulation of timing of transition from vegetative to reproductive phase. These two genes were down regulated after 18 days continuous drought treatment, but the members of regulator *miRNA169s* were both up- and downregulated. This was similar to the results of a previous study in *Arabidopsis* [69], indicating that different *miRNA169* members have complicated interactions in order to accurately regulate gene expression. The interactions within the MIR169 family required further study.

### 3.3. Insights to the Correlation of miRNAs and their Targets

When miRNAs are expressed, they pair to sites within the 3′ untranslated region (UTR) of target mRNAs, and then trigger the translational repression of the mRNA targets [54]. Therefore, we focused on the significant miRNA-target pairs sharing reverse expression patterns, and identified 25 DEmiRs targeting 74 DEGs in 107 miRNA-target pairs. These pairs mainly annotated to “binding”, which is an enriched annotation of drought-responsive genes in senna (*Cassia angustifolia* Vahl) [70]. The comparison of drought-treated roots to control roots yielded six significantly differentially expressed pairs, and we did not identify any pairs with differential expression in leaves. Among these miRNA-target pairs, *ata-miR164c-3p* (downregulated) and its target *comp59407_c0* (*BIPE3*) (upregulated) were notable.

The target *comp59407_c0* (*BIPE3*) was annotated to “ATP binding” in molecular function GO category and “endoplasmic reticulum lumen” in cellular component GO category. It was involved in KEGG pathways “protein export” and “prion diseases” pathways. We found that *ata-miR164c-3p* was downregulated, while its target *BIPE3* was upregulated. *ata-miR164c-3p* was annotated with the following terms: antioxidant activity, cell redox homeostasis, protein-disulfide reductase activity, ATP binding, and endoplasmic reticulum lumen (Table S9). BIPE3 belongs to the HSP protein family which adapts to drought stress [66]. Hence, we inferred that after exposure to drought stress for 18 days *ata-miR164c-3p* expression decreased, causing the accumulation of BIPE3 protein in roots. However, this specific pathway requires further verification through transgenic experiments.

The *ata-miR164c-3p* and *comp59407_c0* (*BIPE3*) pair was also found differentially expressed when comparing expression in leaf and root, regardless of drought treatment. This miRNA–target pair may be regulated differently in root and leaf, or the target may have other epigenetic modifications. Additionally, miRNA *PC-3p-68901_67* and its target *comp50628_c0* (*MNR1*) (a beta-hydroxysteroid dehydrogenase) were both significantly downregulated, indicating that this pair may be involved organ-specific drought adaptation.

Through degradome sequencing, *comp58564_c0* (*CLPC2*), *comp55619_c0* (At1g06650), and *comp58684_c0* (*STP13*) were identified as genes targeted by a relatively high number of miRNAs. Expression of *comp58564_c0* (*CLPC2*) was higher in leaves than in roots, and drought stress led to its downregulation in both organs. On the contrary, *comp55619_c0* (*At1g06650*) had higher expression in roots, and was upregulated under dehydration. As for *comp58684_c0* (*STP13*), it had a different expression pattern in the two organs when faced with drought stress.

ClpC (a heat-inducible protein) appears to be responsible for maintaining homeostasis, and works on plasma membranes where it may control chloroplast preprotein import [71]. Its higher expression in leaves than roots supports this possible function. In addition, the reduced expression of *ClpC2* may be due to the disorder of homeostasis caused by drought stress. *Comp55619_c0* (*At1g06650*) was annotated to “metal ion binding” and “oxidoreductase activity, acting on paired donors, with incorporation or reduction of molecular oxygen and 2-oxoglutarate as one donor, and incorporation of one atom each of oxygen into both donors”, and was associated with the “flavonoid biosynthesis” pathway. A previous study shows a rapid increase in expression level of flavonoid biosynthesis genes under drought stress [72], which is consistent with the expression patterns of *comp55619_c0* (*At1g06650*) in our study. Furthermore, this gene was expressed largely in roots, thus, it might be responsible for the stronger damage tolerance of the roots. Lastly, *comp58684_c0* (*STP13*) showed opposing expression pattern in roots and leaves under dehydration, which was confirmed with both transcriptome sequencing and RT-PCR. STP13, a hexose transporter, functions in improving plant growth and nitrogen use in arabidopsis seedlings and regulates programmed cell death [73,74]. Studies suggest that STP13 is negatively regulated by phosphorylation, and it takes part in the active resorption of hexoses to trigger plant defense reactions with increased energy [75]. Hence, STP13 may play a role in adapting to drought stress in the root through increasing hexose content or other pathways. However, *STP13* had decreased expression in leaves, suggesting that accumulation of soluble sugar in leaves after continuous exposure to drought might inhibit the expression of *STP13* [76].

## 4. Materials and Methods

### 4.1. Plant Material and Water Deficit Treatment

Seedlings from one tiller of *Dactylis glomerata* L. cultivar “Baoxing” (a highly drought-resistant line) were grown in six plastic pots (three seedlings of each pot) in a growth chamber and watered to keep the relative water content (RWC) at 50%. The pots were 16 cm in diameter, 12 cm in ground diameter, and 13 cm in height. The temperature of the growth chamber was set at 22 °C/15 °C (day/night) with humidity at 70%. The photoperiod was set to 14 h/10 h (day/night) under 300 μmol/(m^2^s) light. After two weeks of acclimation, three pots were not irrigated as the drought treatment group, and the other three pots were watered every three days, maintaining soil water content (SWC) at 35%, as the control group. Following previous studies that found that Baoxing orchardgrass shows significant morphological changes after 18 days of drought stress [37,77], we treated samples with an 18-day water-deficit treatment. Under drought conditions, root-sourced signals are transported via the xylem to leaves, and result in reduced water loss and decreased leaf growth [78]. Thus, we chose to focus on both root and leaf to determine drought adaptation patterns after 18 days. Roots and leaves of the control and treatment groups were separately sampled and combined from the three pots. The experiment was conducted in duplicates. We obtained four different kinds of samples: root under control conditions (CK_R), leaf under control conditions (CK_L), root under 18-day drought stress (D18d_R), and leaf under 18-day drought stress (D18d_L). Eight samples (two duplicates for each kind of sample) were frozen in liquid nitrogen once collected, and stored at −80 °C until RNA extraction.

### 4.2. Transcriptome Sequencing and De Novo Assembly Analysis

Total RNA was extracted from root and leaf of control and drought treated orchardgrass using TRIzol reagent (Invitrogen, Carlsbad, CA, USA) according to the manufacturer’s procedure. Total RNA quantity and purity were analyzed using the Bioanalyzer 2100 and RNA 6000 Nano LabChip Kit (Agilent, CA, USA) to ensure that all RNA samples used had a RIN (RNA integrity number) number >7.0 in this study. For each sample, approximately 10 μg of total RNA was subjected to poly(A) mRNA enrichment. Following purification, divalent cations under an elevated temperature were used to fragment the mRNAs. The RNA fragments were reverse-transcribed to construct the cDNA library using the protocol for the mRNA-Seq sample preparation kit (Illumina, San Diego, CA, USA). The average insert size for the paired-end libraries was 300 bp (± 50 bp). Using the vendor’s recommended protocol, paired-end sequencing was run on an Illumina Hiseq2500 System (LC Sciences, San Diego, CA, USA). Low-quality and adapter sequences were trimmed from the total raw reads using the Cutadapt Pipeline [79]. The remaining valid sequencing reads were assembled de novo using Trinity (25 February 2013) under default parameter choices. Expression level of mRNAs were normalized to RPKMs.

The unigenes generated by Trinity were set to sequence alignments to five public protein databases (Swiss-Prot: https://www.uniprot.org/uniprot/?query=*&fil=reviewed%3Ayes, NR: https://ftp.ncbi.nlm.nih.gov/blast/db/FASTA/, KEGG: https://www.kegg.jp/, KOG: ftp://ftp.ncbi.nih.gov/pub/COG/KOG/kyva and Pfam: http://pfam.xfam.org/) and BLASTx (2.2.22) (http://hpc.loni.org/docs/guides/software.php?software=blast). All of the unigenes had homologs in the databases. GENScan1.0 was used for coding sequence prediction.

### 4.3. Small RNA Sequencing and miRNAs Identification

Approximately 1 μg of total RNA was used to generate small RNA libraries in accordance with the TruSeq Small RNA Sample Prep Kit protocol (Illumina, San Diego, CA, USA). We then processed single-end sequencing (36 bp and 50 bp) on an Illumina Hiseq2500 platform at LC-BIO (Hangzhou, China). Data was analyzed following the recommended procedures provided by LC Sciences Service, with modification to predict plant hairpin structures. To remove adapter dimers, junk, low complexity, common RNA families (rRNA, tRNA, snRNA, snoRNA), and repeats, the raw reads were subjected to ACGT101-miR (LC Science, Houston, TX, USA). To identify known miRNAs and novel 3p- and 5p-derived miRNAs, remaining clean reads with lengths 18–25 nucleotides were mapped to specific species precursors in miRBase 21.0 by BLAST. Novel miRNA candidates were defined as unique sequences that mapped to the other arm of known specific species precursor hairpins but opposite to the annotated mature miRNA-containing arm. Subsequently, the remaining sequences were mapped to the other selected species precursors in miRBase 21.0 by BLAST. The mapped pre-miRNAs were analyzed by BLAST against the specific species genomes to determine their genomic locations. Length variation at both 3′ and 5′ ends and one internal mismatch were allowed in the alignment. We defined the above two as known miRNAs. The unmapped sequences were analyzed by BLAST against the specific genomes. Using RNAfold (http://rna.tbi.univie.ac.at/), hairpin RNA structures were predicated from the flank 120 nt sequences. We normalized the expression of miRNAs using a common set of sequences among all samples [80]. Based on normalized deep-sequencing counts, miRNA differential expression was analyzed by selective use of Fisher’s exact test, Chi-squared tests with 2 × 2 or n × n contingency tables, Student’s *t*-test, or ANOVA, according to the experimental design. The significance threshold was set at 0.05.

### 4.4. Degradome Sequencing and Target Identification

Equal amounts of the eight frozen samples were pooled together for RNA extraction. A degradome library was prepared with approximately 20 μg of the pooled total RNA sample. The experimental steps varied considerably from past efforts [26,81,82]. Around 150 ng of poly(A)+ RNA was extracted as input RNA, and was annealed with biotinylated random primers before streptavidin capture of RNA fragments. RNAs containing 5′-monophosphates were subjected to 5′ adaptor ligation, reverse transcription, and PCR. To generate clusters, the purified cDNA library was used on Illumina’s Cluster Station. Libraries were sequenced using the 5′ adapter only, leading to the sequencing of the first 36 nucleotides on an Illumina Hiseq2500 at the LC-BIO (Hangzhou, China).

Raw sequencing reads were analyzed using Illumina’s Pipeline v1.5. The extracted sequencing reads were used to identify potentially cleaved targets using the CleaveLand 3.0 pipeline (http://sites.psu.edu/axtell) [27]. Only perfectly matching alignments for a given read were kept for degradation analysis.

Based on the signature abundance at each occupied transcript position, all of the potentially cleaved transcripts were selected and grouped into the following five categories (0, 1, 2, 3, or 4), using a key previously employed [83,84]:

At the specific position:

Category 0—more than one raw read here, abundance is equal to the maximum on the transcript which only has one maximum;

Category 1—more than one raw read here, abundance is equal to the maximum on the transcript which has over one maximum;

Category 2—more than one raw read here, abundance is more than the median but less than the maximum for the transcript;

Category 3—more than one raw read here, abundance at the position is no more than the median for the transcript;

Category 4—only one raw read here.

Both Target Finder [85] and the degradome density file were used to predict targets of miRNAs.

### 4.5. Verification by qRT-PCR

To validate the mRNA high-throughput sequencing results, RT-qPCR was conducted on a 7300 Real-Time PCR System (Bio-Rad, Hercules, CA, USA) using the same RNA samples described above. Six target genes were chosen for the qRT-PCR analysis. Primers for the target genes were designed using Primer3 (http://frodo.wi.mit.edu/primer3/) and Primer Premier 5.0 software, and primers are listed in Table S1. First-strand cDNAs were synthesized using Prime ScriptRT Master Mix Kit (Takara, Kusatsu, Japan). The qRT-PCR reaction was processed using Bio-Rad CFX96 following the instructions for the SsoFast EvaGreen Supermix Kit (SYBR Green) (Bio-Rad, Hercules, CA, USA). Glyceraldehyde 3-phosphate dehydrogenase (GAPDH) was chosen as the reference gene [86]. The verification procedure and following data analyses was performed following the methods published in a previous study [33]. Three independent replicates were conducted for each PCR reaction. All sequencing results are on the NCBI SRA database under accession number SRP158919 (https://www.ncbi.nlm.nih.gov/sra/SRP158919).

## 5. Conclusions

In summary, we conducted RNA and degradome sequencing analyses of *Dactylis glomerata* L. leaf and root under control and dehydration conditions. We identified 107 candidate miRNA–mRNA regulating pairs (25 DEmiRs with 74 DEGs) involved in drought stress and/or adaptation in leaf and root. Most of the pairs were predicted to be involved in phenylalanine metabolism and biosynthesis, methane metabolism, protein export, and flavonoid biosynthesis. Targets of the miRNAs that were differentially regulated under drought stress were mainly development and stress-associated genes, such as HSP, POT, and naringenin 3-dioxygenase. One pair, ata-miR164c-3p, and its target comp59407_c0 (BIPE3), appeared to be organ-specific. Our findings provide an experimental foundation for exploring the regulatory mechanisms of miRNAs in the plant in adaptation to drought stress.

## Figures and Tables

**Table 1:** Significantly differentially expressed miRNA–target pairs detected in the degradome.

Comparison	miR_Name	Regulation	Gene_ID	Gene Annotation	Regulation
CKL vs. CKR	ata-miR164c-3p	down	comp50213_c0	-	down
	ata-miR164c-3p	down	comp59407_c0	Heat shock 70kda protein 5	down
	PC-3p-68901_67	down	comp50628_c0	11beta-hydroxysteroid dehydrogenase	down
	sbi-MIR396d-p3_2ss2CA18TC	down	comp57297_c0	Proton-dependent oligopeptide transporter	down
	tae-miR319_L-1R+1	down	comp59014_c0	-	down
	ata-miR164c-3p	down	comp59034_c0	-	down
	bdi-miR160e-5p_L+1R-1	up	comp42625_c0	Peroxidase	down
	far-MIR1122-p5_1ss9AG	down	comp58564_c0	ATP-dependent Clp protease ATP-binding subunit clpc	Up
	tae-miR1130b-3p_L+4R-6_1ss6CT	up	comp59143_c0	-	down
D18dR vs. CKR	ata-miR164c-3p	down	comp59407_c0	Heat shock 70kda protein 5	up
	bdi-miR5049-5p_R-2_2ss4TC20TC	down	comp44426_c0	Naringenin 3-dioxygenase	up
	bdi-miR5049-5p_R-2_2ss4TC20TC	down	comp55619_c0	Naringenin 3-dioxygenase	up
	far-MIR1122-p5_1ss9AG	down	comp55619_c0	Proton-dependent oligopeptide transporter	up
	far-MIR1122-p5_1ss9AG	down	comp58684_c0	-	up
	bdi-MIR1135-p3_2ss17CT20GT	up	comp58684_c0	-	up
	sbi-MIR396d-p3_2ss2CA18TC	down	comp57297_c0	-	up

**Table 2:** KEGG annotation of correlation analysis.

Comparison	KEGG ID	Gene_ID	Regulation	KEGG_Name
CK_L vs. CK_R	ko00360	comp42625_c0	down	Phenylalanine metabolism
	ko00680	comp42625_c0	down	Methane metabolism
	ko00940	comp42625_c0	down	Phenylpropanoid biosynthesis
D18d_R vs. CK_R	ko03060	comp59407_c0	up	Protein export
	ko00941	comp55619_c0	up	Flavonoid biosynthesis

## Supplementary Materials

Supplementary materials can be found at http://www.mdpi.com/1422-0067/19/10/3114/s1.

## Abbreviations

GO	Gene ontology
KEGG	Kyoto encyclopedia of genes and genomes
MAPK	Mitogen-activated protein kinase
CDPK	Calcium-dependent protein kinase
miRNA	microRNA
SPL	Squamosa promoter binding protein-like
PARE	Parallel analysis of RNA ends
RACE	5′-rapid amplification of cDNA ends
DEG	Differently expressed gene
AGO	Protein argonaute 2
MYBAS2	MYB-related protein
NAC74	NAC domain-containing protein 74
PERK2	Protein kinase R (PKR)-like endoplasmic reticulum kinase 2
STP13	Sugar transport protein 13
BIPE3	Luminal-binding protein 3
DEmiR	DIFFERENTLY expressed miRNA

## References

[1] Haines A., Kovats R.S., Campbelllendrum D., Corvalan C. (2006). Climate change and human health: Impacts, vulnerability, and mitigation. Public Health.

[2] Schwalm C.R., Williams C.A., Schaefer K., Baldocchi D., Black T.A., Goldstein A.H., Law B.E., Oechel W.C., Kyaw T.P.U., Scott R.L. (2012). Reduction in carbon uptake during turn of the century drought in western North America. Nat. Geosci..

[3] Touma D., Ashfaq M., Nayak M.A., Kao S.C., Diffenbaugh N.S. (2015). A multi-model and multi-index evaluation of drought characteristics in the 21st century. J. Hydrol..

[4] Schwalm C.R., Wrl A., Michalak A.M., Fisher J.B., Biondi F., Koch G., Litvak M., Ogle K., Shaw J.D., Wolf A. (2015). Global patterns of drought recovery. Nature.

[5] Fedoroff N.V., Battisti D.S., Beachy R.N., Cooper P.J., Fischhoff D.A., Hodges C.N., Knauf V.C., Lobell D., Mazur B.J., Molden D. (2010). Radically rethinking agriculture for the 21st century. Science.

[6] Trenberth K.E., Dai A., Schrier G.V.D., Jones P.D., Barichivich J., Briffa K.R., Sheffield J. (2014). Global warming and changes in drought. Nat. Clim. Chang..

[7] Zhu J.K. (2016). Abiotic stress signaling and responses in plants. Cell.

[8] Adachi H., Nakano T., Miyagawa N., Ishihama N., Yoshioka M., Katou Y., Yaeno T., Shirasu K., Yoshioka H. (2015). WRKY transcription factors phosphorylated by MAPK regulate a plant immune NADPH oxidase in *Nicotiana benthamiana*. Plant Cell.

[9] Wang J., Cheng G., Wang C., He Z., Lan X., Zhang S., Lan H. (2017). The bHLH transcription factor CgbHLH001 is a potential interaction partner of CDPK in halophyte *Chenopodium glaucum*. Sci. Rep..

[10] Chen X. (2009). Small RNAs and their roles in plant development. Annu. Rev. Cell Dev. Biol..

[11] Voinnet O. (2009). Origin, biogenesis, and activity of plant microRNAs. Cell.

[12] Zhang B., Wang Q. (2015). MicroRNA-based biotechnology for plant improvement. J. Cell. Physiol..

[13] Akdogan G., Tufekci E.D., Uranbey S., Unver T. (2016). MiRNA-based drought regulation in wheat. Funct. Integr. Genomics.

[14] Shaik R., Ramakrishna W. (2012). Bioinformatic analysis of epigenetic and microRNA mediated regulation of drought responsive genes in rice. PLoS ONE.

[15] Yin F., Gao J., Liu M., Qin C., Zhang W., Yang A., Xia M., Zhang Z., Shen Y., Lin H. (2014). Genome-wide analysis of water-stress-responsive microRNA expression profile in tobacco roots. Funct. Integr. Genom..

[16] Shen J., Xing T., Yuan H., Liu Z., Jin Z., Zhang L., Pei Y. (2013). Hydrogen sulfide improves drought tolerance in *Arabidopsis thaliana* by microRNA expressions. PLoS ONE.

[17] Zhang X., Zou Z., Gong P., Zhang J., Ziaf K., Li H., Xiao F., Ye Z. (2011). Over-expression of microRNA169 confers enhanced drought tolerance to tomato. Biotechnol. Lett..

[18] Thiebaut F., Grativol C., Tanurdzic M., Carnavalebottino M., Vieira T., Motta M.R., Rojas C., Vincentini R., Chabregas S.M., Hemerly A.S. (2014). Differential sRNA regulation in leaves and roots of sugarcane under water depletion. PLoS ONE.

[19] Hamza N.B., Sharma N., Tripathi A., Sanan-Mishra N. (2016). MicroRNA expression profiles in response to drought stress in *Sorghum bicolor*. Gene Expr. Patterns.

[20] Hackenberg M., Gustafson P., Langridge P., Shi B.J. (2015). Differential expression of microRNAs and other small RNAs in barley between water and drought conditions. Plant Biotechnol. J..

[21] Boopathi M.N., Sathish S., Kavitha P., Dachinamoorthy P., Ravikesavan R. (2016). Comparative miRNAome analysis revealed numerous conserved and novel drought responsive miRNAs in cotton (*Gossypium* spp.). Cotton Genom. Genet..

[22] Arshad M., Feyissa B.A., Amyot L., Aung B., Hannoufa A. (2017). MicroRNA156 improves drought stress tolerance in alfalfa (*Medicago sativa*) by silencing SPL13. Plant Sci..

[23] Zhang J., Zhang H., Srivastava A.K., Pan Y., Bai J., Fang J., Shi H., Zhu J.K. (2018). Knockdown of rice microRNA166 confers drought resistance by causing leaf rolling and altering stem xylem development. Plant Physiol..

[24] Pinzón N., Li B., Martinez L., Sergeeva A., Presumey J., Apparailly F., Seitz H. (2017). MicroRNA target prediction programs predict many false positives. Genome Res..

[25] German M.A., Pillay M., Jeong D.H., Hetawal A., Luo S., Janardhanan P., Kannan V., Rymarquis L.A., Kan N., German R. (2008). Global identification of microRNA-target RNA pairs by parallel analysis of RNA ends. Nat. Biotechnol..

[26] Addo-Quaye C., Eshoo T.W., Bartel D.P., Axtell M.J. (2008). Endogenous siRNA and miRNA targets identified by sequencing of the *Arabidopsis* degradome. Curr. Biol..

[27] Addoquaye C., Miller W., Axtell M.J. (2009). CleaveLand: A pipeline for using degradome data to find cleaved small RNA targets. Bioinformatics.

[28] Lin P.C., Lu C.W., Shen B.N., Guan-Zong L., Bowman J.L., Arteaga-Vazquez M.A., Liu L.Y.D., Hong S.F., Chu-Fang L., Su G.M. (2016). Identification of miRNAs and their targets in the liverwort *Marchantia polymorphaby* integrating RNA-seq and degradome analyses. Plant Cell Physiol..

[29] Chen H., Arsovski A.A., Yu K., Wang A. (2016). Genome-Wide investigation using sRNA-seq, degradome-seq and transcriptome-seq reveals regulatory networks of microRNAs and their target genes in doybean during doybean mosaic virus infection. PLoS ONE.

[30] CandarCakir B., Arican E., Zhang B. (2016). SmallRNA and degradome deep sequencing reveals drought-and tissue-specific micrornas and their important roles in drought-sensitive and drought-tolerant tomato genotypes. Plant Biotechnol. J..

[31] Kole C. (2011). Millets and grasses. Wild Crop Relatives: Genomic and Breeding Resources.

[32] Hulten E. (1968). Flora of Alaska and Neighbouring Territories: A Manual of the Vascular Plants.

[33] Feng G., Huang L., Li J., Wang J., Xu L., Pan L., Zhao X., Wang X., Huang T., Zhang X. (2017). Comprehensive transcriptome analysis reveals distinct regulatory programs during vernalization and floral bud development of orchardgrass (*Dactylis glomerata* L.). BMC Plant Biol..

[34] Last L., Widmer F., Fjellstad W., Stoyanova S., Kölliker R. (2013). Genetic diversity of natural orchardgrass (*Dactylis glomerata* L.) populations in three regions in Europe. BMC Genet..

[35] Zhouri L., Kallida R., Shaimi N., Barre P., Volaire F., Gaboun F., Fakiri M. (2016). Evaluation of Cocksfoot (*Dactylis glomerata* L.) Population for Drought Survival and Behavior. Saudi J. Biol. Sci..

[36] Ji Y., Zhang X.Q., Pen Y., Liang X.Y., Huang L.K., Chen L.Z., Li Z., Ma Y.M. (2013). Effects of drought stress on the root growth and photosynthetic characters of *Dactylis glomerata* seedlings. Ying Yong Sheng Tai Xue Bao.

[37] Ji Y., Zhang X., Peng Y., Liang X., Huang L., Ma X., Ma Y. (2014). Effects of drought stress on lipid peroxidation, osmotic adjustment and activities of protective enzymes in the roots and leaves of orchardgrass. Acta Prataculture Sin..

[38] Ding Y., Tao Y., Zhu C. (2013). Emerging roles of microRNAs in the mediation of drought stress response in plants. J. Exp. Bot..

[39] Qin F., Kakimoto M., Sakuma Y., Maruyama K., Osakabe Y., Tran L.S., Shinozaki K., Yamaguchi-Shinozaki K. (2007). Regulation and functional analysis of ZmDREB2A in response to drought and heat stresses in *Zea mays* L.. Plant J..

[40] Ren X., Chen Z., Liu Y., Zhang H., Zhang M., Liu Q., Hong X., Zhu J.K., Gong Z. (2010). ABO3, a WRKY transcription factor, mediates plant responses to abscisic acid and drought tolerance in *Arabidopsis*. Plant J..

[41] Zheng X.N., Chen B., Lu G.J., Han B. (2009). Overexpression of a NAC transcription factor enhances rice drought and salt tolerance. Biochem. Biophys. Res. Commun..

[42] Feng Z.J., Xu S.C., Liu N., Zhang G.W., Hu Q.Z., Gong Y.M. (2018). Soybean TCP transcription factors: Evolution, classification, protein interaction and stress and hormone responsiveness. Plant Physiol. Biochem..

[43] Dai X., Xu Y., Ma Q., Xu W., Wang T., Xue Y., Chong K. (2007). Overexpression of an R1R2R3 MYB gene, OsMYB3R-2, increases tolerance to freezing, drought, and salt stress in transgenic *Arabidopsis*. Plant Physiol..

[44] Ma H.S., Liang D., Shuai P., Xia X.L., Yin W.L. (2010). The salt- and drought-inducible poplar GRAS protein SCL7 confers salt and drought tolerance in *Arabidopsis thaliana*. J. Exp. Bot..

[45] Kantar M., Lucas S.J., Budak H. (2011). MiRNA expression patterns of *Triticum dicoccoides* in response to shock drought stress. Planta.

[46] Pasini L., Bergonti M., Fracasso A., Marocco A., Amaducci S. (2014). Microarray analysis of differentially expressed mRNAs and miRNAs in young leaves of sorghum under dry-down conditions. J. Plant Physiol..

[47] Zhang N., Yang J., Wang Z., Wen Y., Wang J., He W., Liu B., Si H., Wang D. (2014). Identification of novel and conserved microRNAs related to drought stress in potato by deep sequencing. PLoS ONE.

[48] Stewart A.V., Ellison N.W. (2011). Dactylis. Wild Crop Relat. Genomic Breed. Resour..

[49] Chen Z.H., Bao M.L., Sun Y.Z., Yang Y.J., Xu X.H., Wang J.H., Han N., Bian H.W., Zhu M.Y. (2011). Regulation of auxin response by miR393-targeted is involved in normal development in *Arabidopsis*. Plant Mol. Biol..

[50] Windels D., Vazquez F. (2011). MiR393: Integrator of environmental cues in auxin signaling?. Plant Signal. Behav..

[51] Gao P., Bai X., Yang L., Lv D., Pan X., Li Y., Cai H., Ji W., Chen Q., Zhu Y. (2011). Osa-MIR393: A salinity- and alkaline stress-related microRNA gene. Mol. Biol. Rep..

[52] Chen Z., Hu L., Han N., Hu J., Yang Y., Xiang T., Zhang X., Wang L. (2015). Overexpression of a miR393-resistant form of transport inhibitor response protein 1 (mTIR1) enhances salt tolerance by increased osmoregulation and Na+ exclusion in *Arabidopsis thaliana*. Plant Cell Physiol..

[53] Bai B., Bian H., Zeng Z., Hou N., Shi B., Wang J., Zhu M., Han N. (2017). MiR393-mediated auxin signaling regulation is involved in root elongation inhibition in response to toxic aluminum stress in barley. Plant Cell Physiol..

[54] Reinhart B.J., Weinstein E.G., Rhoades M.W., Bartel B., Bartel D.P. (2002). MicroRNAs in plants. Genes Dev..

[55] Fang Y., Xie K., Xiong L. (2014). Conserved miR164-targeted NAC genes negatively regulate drought resistance in rice. J. Exp. Bot..

[56] Wang Y., Lin L., Sha T., Liu J., Zhang H., Hui Z., Jia G., Diao X. (2016). Combined small RNA and degradome sequencing to identify miRNAs and their targets in response to drought in foxtail millet. BMC Genet..

[57] Kansal S., Devi R.M., Balyan S.C., Arora M.K., Singh A.K., Mathur S., Raghuvanshi S. (2015). Unique miRNome during anthesis in drought-tolerant indica rice var. Nagina 22. Planta.

[58] Phookaew P., Netrphan S., Sojikul P., Narangajavana J. (2014). Involvement of miR164- and miR167-mediated target gene expressions in responses to water deficit in cassava. Biol. Plant..

[59] Song C.P., Agarwal M., Ohta M., Guo Y., Halfter U., Zhu J.K. (2005). Role of an *Arabidopsis* AP2/EREBP-type transcriptional repressor in abscisic acid and drought stress responses. Plant Cell.

[60] Xiang Y., Tang N., Du H., Ye H., Xiong L. (2008). Characterization of OsbZIP23 as a key player of the basic leucine zipper transcription factor family for conferring abscisic acid sensitivity and salinity and drought tolerance in rice. Plant Physiol..

[61] Hussain R.M., Ali M., Feng X., Li X. (2017). The essence of NAC gene family to the cultivation of drought-resistant soybean (*Glycine max* L. Merr.) cultivars. BMC Plant Biol..

[62] Abe H., Yamaguchishinozaki K., Urao T., Iwasaki T., Hosokawa D., Shinozaki K. (1997). Role of *Arabidopsis* MYC and MYB homologs in drought- and abscisic acid-regulated gene expression. Plant Cell.

[63] Cho E.K., Hong C.B. (2006). Over-expression of tobacco NtHSP70-1 contributes to drought-stress tolerance in plants. Plant Cell Rep..

[64] Sakuma Y., Maruyama K., Osakabe Y., Qin F., Seki M., Shinozaki K., Yamaguchishinozaki K. (2006). Functional analysis of an *Arabidopsis* transcription factor, DREB2A, involved in drought-responsive gene expression. Plant Cell.

[65] Hansen T.B., Wiklund E.D., Bramsen J.B., Villadsen S.B., Statham A.L., Clark S.J., Kjems J. (2011). MiRNA-dependent gene silencing involving Ago2-mediated cleavage of a circular antisense RNA. EMBO J..

[66] Cho E.K., Choi Y.J. (2009). A nuclear-localized HSP70 confers thermoprotective activity and drought-stress tolerance on plants. Biotechnol. Lett..

[67] Cao H.Q., Jiang S.L., Huang C.M., Deng Z.N., Wu K.C., Xu L., Luo H.B., Lu Z., Wei Y.W. (2017). Cloning of ATP binding protein gene in sugarcane and its expression characteristics under drought stress. J. South. Agric..

[68] Buriani G., Mancini C., Benvenuto E., Baschieri S. (2011). Plant heat shock protein 70 as carrier for immunization against a plant-expressed reporter antigen. Transgenic Res..

[69] Du Q., Zhao M., Gao W., Sun S., Li W.X. (2017). MicroRNA/microRNA* complementarity is important for the regulation pattern of NFYA5 by miR169 under dehydration shock in *Arabidopsis*. Plant J..

[70] Mehta R.H., Ponnuchamy M., Kumar J., Reddy N.R.R. (2017). Exploring drought stress-regulated genes in senna (*Cassia angustifolia* Vahl.): A transcriptomic approach. Funct. Integr. Genomics.

[71] Sjögren L.L., Tanabe N., Lymperopoulos P., Khan N.Z., Rodermel S.R., Aronsson H., Clarke A.K. (2014). Quantitative analysis of the chloroplast molecular chaperone ClpC/Hsp93 in *Arabidopsis* reveals new insights into its localization, interaction with the Clp proteolytic core, and functional importance. J. Biol. Chem..

[72] Ma D., Sun D., Wang C., Li Y., Guo T. (2014). Expression of flavonoid biosynthesis genes and accumulation of flavonoid in wheat leaves in response to drought stress. Plant Physiol. Biochem..

[73] Schofield R.A., Bi Y.M., Kant S., Rothstein S.J. (2009). Over-expression of STP13, a hexose transporter, improves plant growth and nitrogen use in *Arabidopsis thaliana* seedlings. Plant Cell Environ..

[74] Nørholm M.H.H., Nour-Eldin H.H., Brodersen P., Mundy J., Halkier B.A. (2006). Expression of the *Arabidopsis* high-affinity hexose transporter STP13 correlates with programmed cell death. FEBS Lett..

[75] Lemonnier P., Gaillard C., Veillet F., Verbeke J., Lemoine R., Coutosthévenot P., La C.S. (2014). Expression of *Arabidopsis* sugar transport protein STP13 differentially affects glucose transport activity and basal resistance to *Botrytis cinerea*. Plant Mol. Biol..

[76] Mostajeran A., Rahimieichi V. (2009). Effects of drought stress on growth and yield of rice (*Oryza sativa* L.) cultivars and accumulation of proline and soluble sugars in sheath and blades of their different ages leaves. Am. Eurasian J. Agric. Environ. Sci..

[77] Ji Y., Zhang X., Peng Y., Huang L., Liang X., Wang K., Yin G., Zhao X. (2015). Osmolyte accumulation, antioxidant enzyme activities and gene expression patterns in leaves of orchardgrass during drought stress and recovery. Grassl. Sci..

[78] Schachtman D.P., Goodger J.Q.D. (2008). Chemical root to shoot signaling under drought. Trends Plant Sci..

[79] Martin M. (2011). Cutadapt removes adapter sequences from high-throughput sequencing reads. EMBnet J..

[80] Li X., Shahid M.Q., Wu J., Wang L., Liu X., Lu Y. (2016). Comparative small RNA analysis of pollen development in autotetraploid and diploid rice. Int. J. Mol. Sci..

[81] Addoquaye C., Snyder J.A., Yong B.P., Li Y.F., Sunkar R., Axtell M.J. (2009). Sliced microRNA targets and precise loop-first processing of MIR319 hairpins revealed by analysis of the *Physcomitrella patens* degradome. RNA.

[82] Ma Z.R., Coruh C., Axtell M.J. (2010). *Arabidopsis lyrata* small RNAs: Transient miRNA and small interfering RNA loci within the *Arabidopsis* genus. Plant Cell.

[83] Xu X., Yin L., Ying Q., Song H., Xue D., Lai T., Xu M., Shen B., Wang H., Shi X. (2013). High-throughput sequencing and degradome analysis identify miRNAs and their targets involved in fruit senescence of *Fragaria ananassa*. PLoS ONE.

[84] Yang X., Wang L., Yuan D., Lindsey K., Zhang X. (2013). Small RNA and degradome sequencing reveal complex miRNA regulation during cotton somatic embryogenesis. J. Exp. Bot..

[85] Fahlgren N., Howell M.D., Kasschau K.D., Chapman E.J., Sullivan C.M., Cumbie J.S., Givan S.A., Law T.F., Grant S.R., Dangl J.L. (2007). High-throughput sequencing of *Arabidopsis* microRNAs: Evidence for frequent birth and death of miRNA genes. PLoS ONE.

[86] Reid K.E., Olsson N., Schlosser J., Peng F., Lund S.T. (2006). An optimized grapevine RNA isolation procedure and statistical determination of reference genes for real-time RT-PCR during berry development. BMC Plant Biol..

